# Supplementary material for: Subset of DN Memory B Cells Expressing Low Levels of Inhibitory Receptor BTLA Is Enriched in SLE Patients
Source: Cells. 2024 Dec 13;13(24):2063. doi: 10.3390/cells13242063 (PMC11674271; doi:10.3390/cells13242063)
Supplement: Supplementary file 1 [file cells-13-02063-s001.zip › cells-3302280-supplementary.pdf]

cells

Supplementary Matrials

Subset of DN Memory B Cells Expressing Low Levels of Inhibitory Receptor BTLA is Enriched in SLE Patients

Lucie Aubergeon [1], Renaud Felten [1,2], Jacques-Eric Gottenberg [1,2], Hélène Dumortier [1] and Fanny Monneaux [1,*]

[1] Immunology, Immunopathology and Therapeutic Chemistry, Institute of Molecular and Cellular Biology, CNRS UPR3572, 67084 Strasbourg, France
[2] Rheumatology Department, National Reference center for Autoimmune Diseases, Strasbourg University Hospital, Strasbourg, 67000, France
* Correspondence: f.monneaux@ibmc-cnrs.unistra.fr

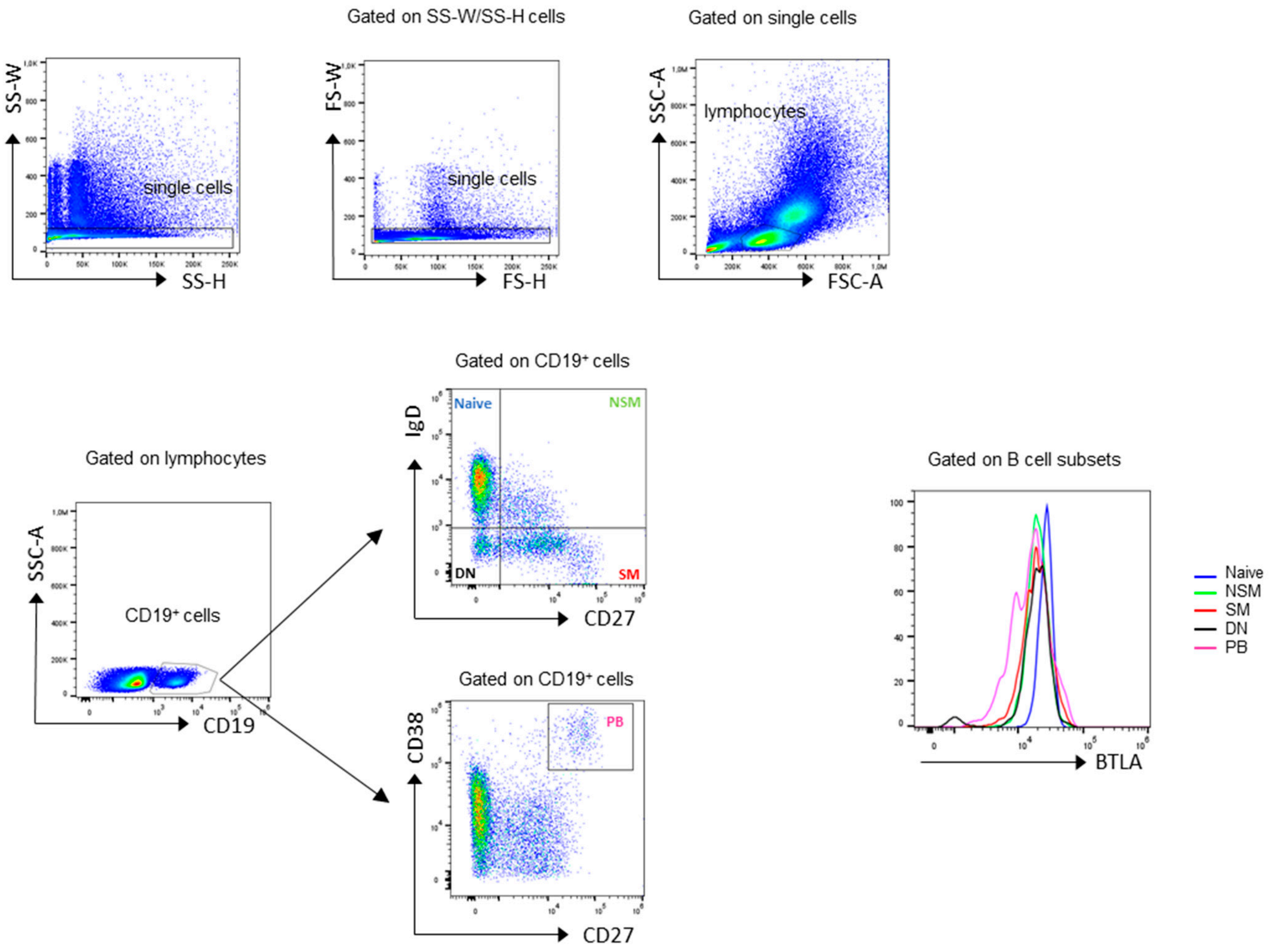

Supplementary Figure S1. Flow cytometry gating strategy of B cell subsets defined by CD19, IgD, CD27, CD38.

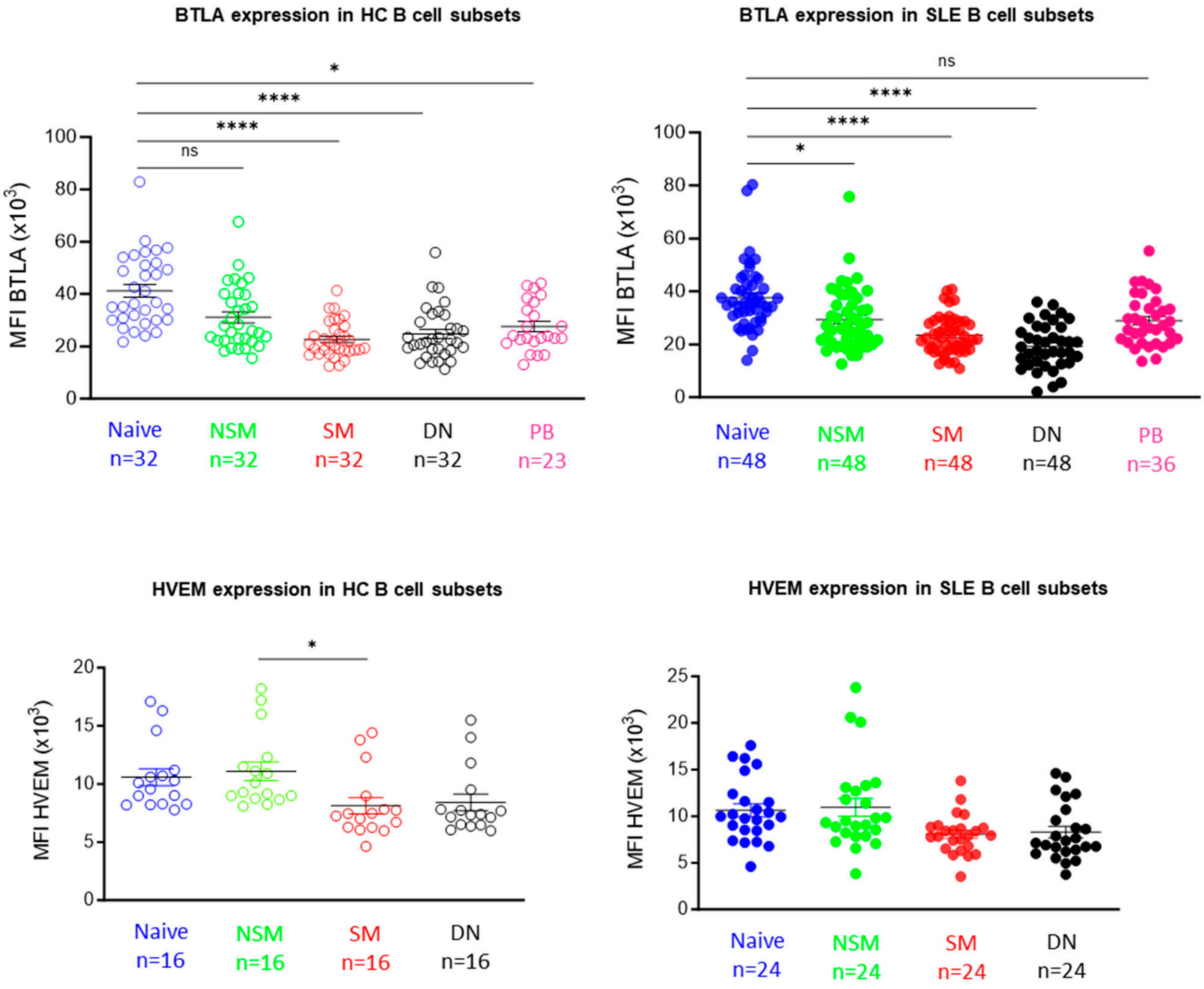

Supplementary Figure S2. BTLA and HVEM expression on B cells from HC and SLE patients. B cell subsets were identified by cytometry among B cells ($CD19^{+}$) as naive B cell ($CD19^{+}IgD^{+}CD27^{-}$), non-switched memory B cells (NSM; $CD19^{+}IgD^{+}CD27^{+}$), switched memory B cells (SM; $CD19^{+}IgD^{-}CD27^{+}$), double negative memory B cells (DN; $CD19^{+}IgD^{-}CD27^{-}$) and plasmablasts (PB; $CD19^{+}CD27^{hi}CD38^{hi}$). Comparison of BTLA (upper panel) and HVEM (lower panel) expression on B cell subsets in HC and SLE patients. Results are expressed as MFI and horizontal lines represent the mean of BTLA or HVEM expression. *p<0.05, ****p<0.0001; Kruskal-Wallis.

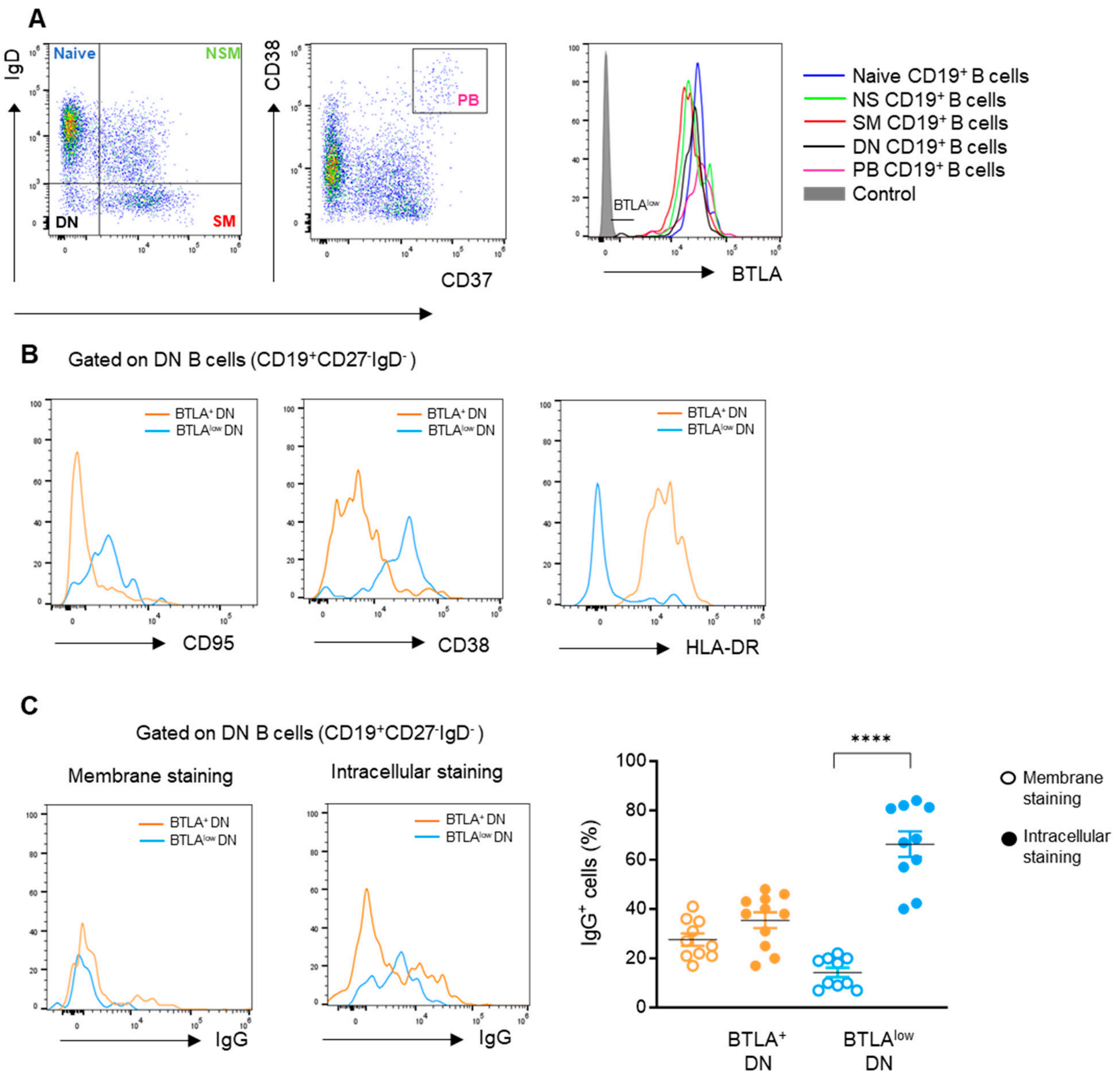

Supplementary Figure S3. Characteristics of BTLA^{low} DN B cells from HC. (A) B cell subsets were identified by cytometry among B cells (CD19^{+}) as naive B cell (CD19^{+}IgD^{+}CD27^{-}), non-switched memory B cell (NSM; CD19^{+}IgD^{+}CD27^{+}), switched memory B cells (SM; CD19^{+}IgD^{-}CD27^{+}), double negative memory B cells (DN; CD19^{+}IgD^{-}CD27^{-}) and plasmablasts (PB; CD19^{+}CD27^{hi}CD38^{hi}) and a representative histogram of BTLA staining on DN memory B cells from a HC. (B) Representative histogram of CD95, CD38 and HLA-DR expression in BTLA^{+} and BTLA^{low} DN B cells in HC. (C) Membrane and intracellular expression of IgG in BTLA^{+} and BTLA^{low} DN B cells from HC (n = 10). Results are expressed as mean ± SEM. ****$p < 0.0001$, Mann-Whitney tests.

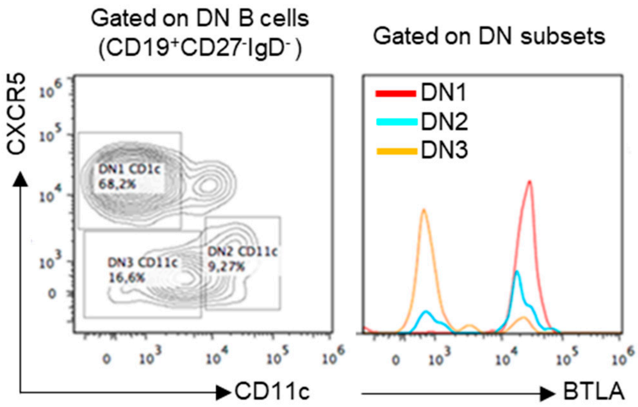

Supplementary Figure S4. DN3 B cells from HC express lower levels of BTLA than other DN B cell subsets. Gating strategy to discriminate DN1, DN2 and DN3 B cell subsets and an example of BTLA staining on each subset are represented.

Disclaimer/Publisher's Note: The statements, opinions and data contained in all publications are solely those of the individual author(s) and contributor(s) and not of MDPI and/or the editor(s). MDPI and/or the editor(s) disclaim responsibility for any injury to people or property resulting from any ideas, methods, instructions or products referred to in the content.
